# A Guild-Based Protocol to Target Potential Natural Enemies of *Philaenus spumarius* (Hemiptera: Aphrophoridae), a Vector of *Xylella fastidiosa* (Xanthomonadaceae): A Case Study with Spiders in the Olive Grove

**DOI:** 10.3390/insects11020100

**Published:** 2020-02-03

**Authors:** Jacinto Benhadi-Marín, María Villa, Luís F. Pereira, Isabel Rodrigues, Marina Morente, Paula Baptista, José Alberto Pereira

**Affiliations:** 1 Centro de Investigação de Montanha (CIMO), ESA, Instituto Politécnico de Bragança, Campus de Santa Apolónia, 5300-253 Bragança, Portugal; mariavillaserrano@gmail.com (M.V.); luispereira17@live.com.pt (L.F.P.); irodrigues@ipb.pt (I.R.); pbaptista@ipb.pt (P.B.); jpereira@ipb.pt (J.A.P.); 2 Instituto de Ciencias Agrarias, Consejo Superior de Investigaciones Científicas, ICA-CSIC, Calle Serrano 115 dpdo, 28006 Madrid, Spain; mmorente@ica.csic.es

**Keywords:** functional response, hunting strategy, quick decline syndrome, spittlebug, predators, biological control

## Abstract

The olive grove is a key landscape across the Mediterranean basin. This agroecosystem is threatened by *Xylella fastidiosa*, the causal agent of the olive tree quick decline syndrome, *Philaenus spumarius* being the main vector. A way to limit pest populations relies on the use of biological control agents such as arthropods. Among them, spiders are generalist predators with different hunting strategies that feed mostly on insects and can contribute to limit pests. In this work, field and laboratory data were used to provide a protocol aiming to facilitate the selection of species of spiders among different guilds that could represent potential natural enemies of *P. spumarius*. Sampling of spiders was conducted in olive groves in northeastern Portugal. Two species, namely the orb-weaver *Araniella cucurbitina* and the ambusher *Synema globosum*, were selected according to the dominant guilds of spiders inhabiting the olive crop. We tested the differences of potential predatory efficiency using classical functional response tests with *P. spumarius* as prey. A type-II functional response was found for *A. cucurbitina*, whereas a type-I response was found for *S. globosum*. This difference uncovers a different potential efficiency among the two species as natural enemies of *P. spumarius* with relevant implications at high prey density in the field. A conceptual workflow to follow the fieldwork and selection of species for further work (i.e., laboratory assays) is provided and discussed. Standardized methods regarding the assessment of the suitability and efficiency of potential natural enemies are essential for the integration of results at different geographical extents and crops. Selecting functional counterparts such as different species of predators occurring at different locations that use the same prey (e.g., a pest) in the same way (e.g., hunting strategy) would facilitate developing biological control schemes.

## 1. Introduction

The olive grove agroecosystem represents a high economic, social and cultural landscape along the Mediterranean basin where 95% of worldwide olive oil is produced [1]. *Xylella fastidiosa* Wells et al. 1986 (Xanthomonadales: Xanthomonadaceae) is a xylem-limited Gram-negative gammaproteobacterium that affects several economically important crops, such as the olive tree, being the causal agent of the olive tree quick decline syndrome [2,3]. The main vector of *X. fastidiosa* in Europe is the meadow spittlebug, *Philaenus spumarius* L. 1758 (Hemiptera: Aphrophoridae) [4]. *Philaenus spumarius* is a polyphagous and widespread insect whose nymphs feed on herbaceous plants, molting within a mass of foam composed by clusters of protein and mucopolysaccharide-containing bubbles [5,6]. The adults remain in the field after the last molt (or emergence) and, as succulent materiel for feeding declines, they gradually disperse to other plants, such as trees and shrubs [4,7]. The acquisition and retention of the bacterium occurs in the foregut cuticle [8,9]. *Philaenus spumarius* remain infective throughout its adult stage being able to transmit *X. fastidiosa* from infected to uninfected olive trees [4]. 

The control of *X. fastidiosa* relies on an approach based on prevention using resistant varieties, cultural and hygienic measures and chemical and biological vector control [10]. Regarding the latter method, although Whittaker [11] pointed out that predation could not be relevant in terms of mortality of meadow spittlebugs, some predators among vertebrates (e.g., birds and frogs) and arthropods (e.g., Diptera, Coleoptera, Hymenoptera, and Opiliones), as well as few parasitoids (e.g., Diptera and Hymenoptera), have been reported in the literature [12]. Moreover, *Zelus renardii* Kolenati 1857 (Heteroptera: Reduviidae) was proposed as a potential biocontrol agent of *P. spumarius* in olive groves [13]. This diversity could be promising for natural limitation purposes, although the use of biological control agents implies not only risks [14], but several steps such as finding, collecting, and shipping, quarantines, safety of the introduction in the case of exotic species, and successful field colonization [15], that if not achieved could make the approach unsuccessful or even threat the ecosystem stability. Indeed, pest limitation strategies based on a biological control agent that include population releasing or habitat management within the crop to enhance its fitness such as sowing host plants or providing artificial shelter [16,17], largely depend on detailed knowledge of the biology of the organism. Moreover, the selection of a cosmopolitan organism with a broad niche (e.g., broad host range) could facilitate the maintenance of the released population in the field or the enhancement of a naturally occurring population [18].

A functional approach to the study of pest regulation based on guilds (i.e., different groups of organisms using a resource in the same way) could facilitate decision making towards the selection of a potential biological control agent. In this sense, the functional point of view can rely on foraging traits. In fact, Dainese et al. [19] demonstrated that biological control can be enhanced by complementarity among guilds of natural enemies such as parasitoids and ground-dwelling spiders. Among arthropods, spiders are generalist predators that feed mostly on insects and, for a long time, have been considered potential natural enemies of pests due to their ubiquity and abundance [20,21,22]. Due to the potential of spiders as agents of pest limitation, attention has been specifically paid to the diversity of guilds in crops according to ecological traits, such as their hunting strategies [23]. Although different guilds of spiders according to hunting strategies can influence the food webs through specific predator-prey interactions, the functional traits could not necessarily represent true functional differences [24]. In addition, information about key aspects of the biological control potential of spiders, such as prey preference, feeding rate, and fitness [25], is still scarce and there is a systematic lack of standardized methods for research.

In this work, our background on the diversity and dynamics of the araneofauna of selected olive crops in Northeastern Portugal was used to propose a protocol aiming at facilitating the task of selecting species of spiders that could represent potential natural enemies of *P. spumarius*. A functional point of view was followed based on guilds derived from hunting strategies. Accordingly, the objectives of this work were to: (1) assess the functional response of two widespread species of spiders in the study area that consistently inhabit selected olive groves, (2) determine the dominance of the guilds these species represent in the field during the raising of *P. spumarius* populations, and (3) use the gathered information to define a workflow from the field to the laboratory relying on a low-effort field sampling protocol.

## 2. Materials and Methods 

### 2.1. Study Area

The fieldwork was conducted near Mirandela (Northeastern Portugal), a Mediterranean area located within the region of Trás-os-Montes with a mean annual rainfall of 776 mm and a mean annual temperature of 13.8 °C [26]. Within this area, where the olive agroecosystem represents an important crop, two adjacent olive groves were selected, hereafter Cedães 1 and Cedães 2, whereas Cedães 1 (41° 29′ 15.63″ N, 7° 7′ 33.58″ O) is a sloped grove at 353 m altitude with an area of 8.90 ha. Cedães 2 (41° 29′ 16.86″ N, 7° 7′ 51.50″ O) is a flat grove at 342 m altitude with an area of 4.01 ha. Both groves present an average distance between rows and inter-rows of 7 m.

### 2.2. Functional Response Assay

#### 2.2.1. Selection of Predator and Prey Species

Two species of spiders were initially selected as predator models based on the consistence of the occurrence of their guild on the study area according to our previous information (data not published) and their cosmopolitan features. The selected species were the generalist orb-weaver *Araniella cucurbitina* (Clerck, 1757) (Araneidae) and e generalist ambusher *Synema globosum* (Fabricius, 1775) (Thomisidae). The specimens of *S. globosum* were captured in early May 2017 by inspecting flowers of *Cistus ladanifer* L. (Malvales: Cistaceae) in a semi-natural shrubland area adjacent to Cedães 1, whereas the specimens of *A. cucurbitina* were captured by inspecting low sized pine trees around Bragança (41° 50′ 19″ N; 6° 44′ 49″ W) during the night.

Once captured, spiders of both species were immediately transported to the laboratory, placed individually into Petri dishes (7 cm in diameter) and maintained in a rearing room at 21 °C, 70 ± 5% relative humidity, and a photoperiod of 16:8 (L:D) h. Since all the captured individuals corresponded to immatures, the spiders were fed ad libitum with adults of the medfly *Ceratitis capitata* (Wiedemann, 1824) (Diptera: Tephritidae) untill adulthood. Individuals of *C. capitata* were obtained from laboratory rearing maintained at the School of Agriculture of Bragança (method described in Dinis et al. [27]).

After the establishment of the adult spider colony in laboratory, spiders were starved for seven days (i.e., all the spiders were seven-day adults when used in the trials) to homogenize the hunger level. Then, adult individuals of *P. spumarius* were collected massively on the field at Cedães 1 and transported to the laboratory to be immediately used as prey for the functional response assay. Males and females of *P. spumarius* were indistinctly used as preys.

#### 2.2.2. Laboratory Trials

The functional response (i.e., the relationship between the number of preys killed or eaten by one predator during a certain time and the prey density) of each spider species fed on adults of *P. spumarius* was assessed in experimental arenas. Five arenas were used for each of six initial prey densities (N = 3, 5, 10, 15, 25 and 40 individuals). Only female spiders were used as predators whereas both sexes of *P. spumarius* were used as preys. Males and females of *P. spumarius* were randomly mixed and placed within the arenas (i.e., no specific sex rate was considered). Each arena was built using a round plastic cage (7.7 cm in diameter and 4.3 cm height) covered with the lid of a glass Petri dish (9 cm in diameter) [28,29].

Assays were conducted at the same rearing room and conditions described in Section 2.2.1. Each spider was allowed to feed during 24 h and the number of prey killed in each arena was recorded. Since the functional response assays lasted only 24 h and we did not observe mortality of *P. spumarius* under the referred conditions during this period in previous tests, no parallel control tests of mortality were conducted. Each arena was carefully inspected to ensure that 100% of individuals of *P. spumarius* were alive before placing the spiders, so spittlebug mortality after 24 h was assumed to be caused by the spiders regardless of consumption (i.e., dead individuals because of feeding or wasteful killing were indistinctly considered as killed).

### 2.3. Field Sampling Scheme

#### Sampling of Araneae

Spiders were captured at three sampling dates in 2019, 22 March, 30 April, and 26 May coinciding with the appearance of foams, development of nymphs, and the emergence of adults *of P. spumarius* in the study area, respectively (P1, P2, and P3 hereafter). In each grove (Cedães 1 and Cedães 2) and sampling date, 10 sites, each one of 2 m^2^ were randomly selected within areas where incipient foams were detected. Sampling sites were at least 20 m apart and were selected along five parallel transects throughout the grove (i.e., 2 sampling points per transect). At each selected area, spiders were captured both across the soil surface and the herbaceous layer during 15 minutes using a sweep net, a mouth aspirator, and by hand. Each site represented a single sample (i.e., a total of 20 samples per sampling date). The collected samples were preserved in situ in ethanol 70%. Spiders were identified to species using specific keys [30] and families were grouped into guilds according to hunting habits following Uetz et al. [23] and Cardoso et al. [31].

### 2.4. Data Analysis

#### 2.4.1. Functional Response Assay

All the data analyses regarding the functional responses were performed in R [32] using the *frair* package [33]. The type of the functional response (i.e., type-I, type-II, or type-III) was determined using a linear regression (to assess the fitting to a type-I response) and then a logistic regression (to assess the fitting to a type-II or type-III response) of the proportion of preys consumed as a function of the number of preys offered using the *frair_test* function. A negative linear coefficient means a better adjustment to type-II, whereas a positive linear coefficient and a negative quadratic coefficient imply that the data fit a type-III functional response [34,35]. According to the type of functional response found for each species of spider, the corresponding curves were fitted using a linear type-I functional response

(1) $$N_{e}=\;aN_{0}T\;\;\;$$

Or the Rogers’s type-II decreasing prey function (for experiments without prey replacement, i.e., prey depletion) [36]:

(2) $$N_{e}=\;N_{0}\left\{1-\;e^{\left[a\left(N_{e\;}T_{h}-\;T\right)\right]}\right\}\;\;$$

where for both models *N_e_* represents the number of prey killed, *N*_0_ is the initial prey density, *a* is the attack rate (searching efficiency per time), *T_h_* is the handling time, and T the time of the experiment. The fact that *N_e_* appears in both sides of the Equation (2) is due to lacking of prey replacement during the experiment and is solved using the Lambert’s transcendental equation [37]. In the case of a type-II functional response, the maximum attack rate (i.e., the maximum number of prey that can be attacked by the predator during *T*) was calculated as a point estimator as *T*/*T_h_*. The functional response parameters, attack rate, and handling time, were estimated using the *frair_fit* function. After sketching the curves, the 95% confidence interval for each one was generated by bootstrapping (999 replicates) using the *frair_boot* and *drawpoly* functions.

#### 2.4.2. Field Sampling Scheme

Data analyses were conducted in R [32]. Firstly, a series of individual-based accumulation curves (i.e., the expected number of collected species as the number of captured individuals increases) for the recorded species and guilds were calculated for each sampling date to assess the quality of the inventory. For this, the function *rarefaction* from [38] based on the function *rarefy* from package *vegan* [39] was used. Then, the abundance among guilds of spiders (i.e., count data) was compared for each sampling date using generalized estimating equations (GEE) with Poisson distribution, an extension of generalized linear models (GLM) [40,41] followed by a posthoc Tukey test. In all cases, the correlation structure used was “exchangeable” (i.e., a single correlation parameter, ρ) and the grove was used for clustering.

## 3. Results

### 3.1. Functional Response Laboratory Assay

**Figure 1:**
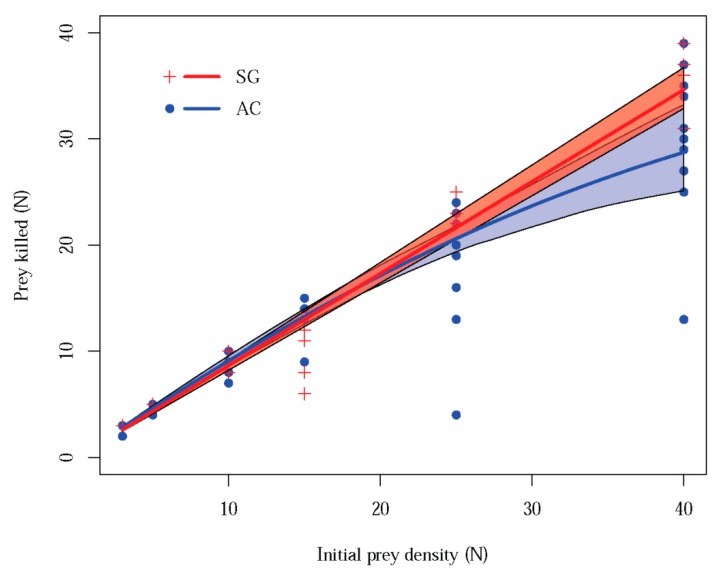
Functional response models obtained for *Araniella cucurbitina* (orb weaver) (AC) and *Synema globosum* (ambusher) (SG) fed on adults of *Philaenus spumarius* during 24 h no-choice experiments. Thick lines represent the fitted values of data and the bands that surround them represent the limits of the 95% confidence interval of the curves.

**Figure 2:**
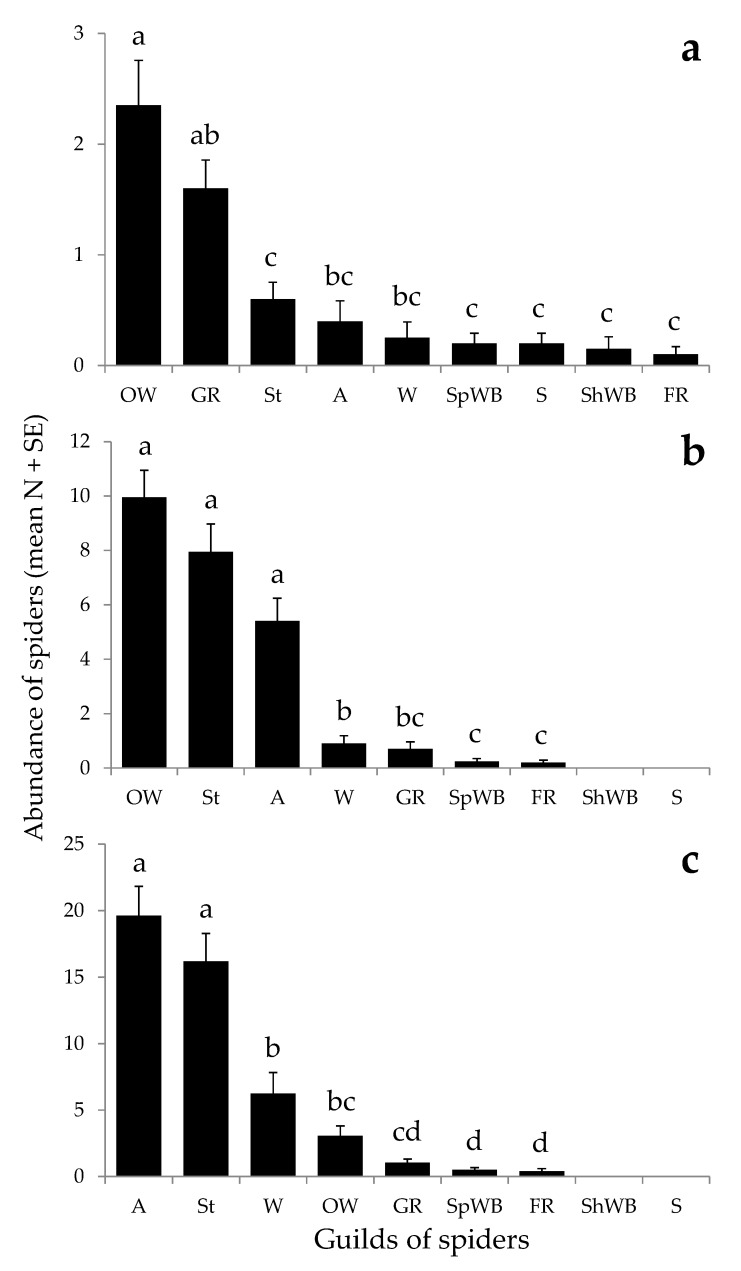
Abundance of guilds of spiders found in the studied olive groves at three sampling periods: (**a**) appearance of foams (P1), (**b**) development of nymphs (P2), and (**c**) emergence of adults of *Philaenus spumarius* (P3). Different letters above bars indicate statistically significant differences (*p* < 0.05). A: Ambushers; St: Stalkers; W: Wandering sheet/tangle weavers; OW: Orb-weavers; GR: Ground runners; SpWB: Space web builders; FR: Foliage runners; ShWB: Sheet web builders; S: Specialists. Note that scales differ on *y* axes.

A type-I functional response was found for *S. globosum* (Figure 1) with an attack rate (*a*) = 0.036 (SE = 0.001; Z = 56.085; *p* < 0.01) whereas the logistic regression indicated a type-II functional response for *A. cucurbitina* (estimate = −0.041; SE = 0.0070942; Z = −5.83; *p* < 0.01) (Figure 2) with an attack rate (*a*) = 0.123 (SE = 0.012; Z = 10.17; *p* < 0.01), a handling time (*T_h_*) = 0.475 (SE = 0.061; Z = 7.85; *p* < 0.01), and a maximum attack rate (*T*/*T_h_*) = 50.52. Both species of spiders killed approximately 30 individuals of *P. spumarius* at the maximum initial prey density (i.e., *N*_0_ = 40). Apart from the type of functional response, in none of the cases the asymptote was reached and the resulting regression for each species did not significantly differ within the range of initial prey densities regarding the overlapping of the 95% confidence intervals (Figure 1). 

### 3.2. Field Sampling Scheme

In total, 1578 spiders were captured encompassing 17 families, nine functional groups and representing at least 45 species (Table 1). The total abundance increased according to the sampling period from 117 to 508, and 953 individuals at P1, P2, and P3, respectively.

**Figure 3:**
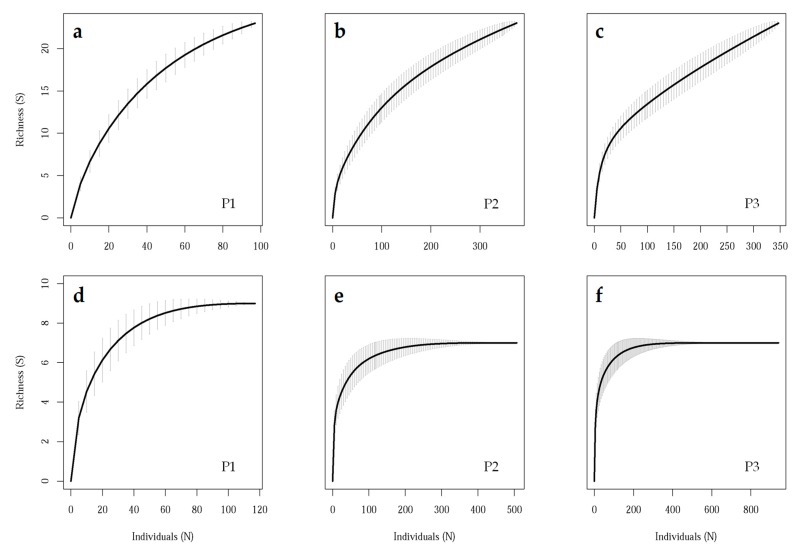
(**a**–**c**) Species accumulation curves and (**d**–**f**) guild accumulation curves at three sampling periods: appearance of foams, development of nymphs, and emergence of adults of *Philaenus spumarius*, respectively, in the studied olive groves. S: Number of species (upper panels) and guilds of spiders (lower panels).

The individual-based accumulation curves were far from reaching a plateau in the case of species for each sampling period (Figure 3a–c), whereas a clear asymptote was reached for functional groups at approximately eight, seven, and seven guilds for P1, P2, and P3, respectively (Figure 3d–f).

The most abundant species among the adult specimens were *Mangora acalypha* (Walckenaer, 1802) in P1 and P2, and *Oxyopes nigripalpis* Kulczyński, 1891 in P3 (Table 1). The dominant families were Araneidae and Gnaphosidae in P1, Araneidae and Oxyopidae in P2, and Thomisidae and Oxyopidae in P3 (Table 1). The dominant guilds were the orb-weavers and ground runners, orb-weavers and stalkers, and ambushers and stalkers in P1, P2, and P3, respectively (Figure 3). The overall abundance among guilds significantly differed within each sampling period (χ^2^ = 121.31; *df* = 8; *p* < 0.01 in P1, χ^2^ = 373.40; *df* = 6; *p* < 0.01 in P2, and χ^2^ = 630.90; *df* = 6; *p* < 0.01 in P3). However, no statistically significant differences were found between the two most abundant guilds at each sampling period (Figure 2).

## 4. Discussion

In this work, we gathered data from both the laboratory and the field to assess the predatory potential of *P. spumarius* by two species of spiders and propose a fast and easy protocol to target potential natural enemies of *P. spumarius* among spiders. The field part was focused on finding dominant guilds rather than on a most abundant species approach, thus reducing the importance of the difference between the naturally occurring spider assemblages at different regions considering functional counterparts (i.e., different species with an equivalent role within the assemblage). Our results support the use of a low field sampling effort (*n* = 20), since according to the accumulation curves for the three sampling periods, all guilds inhabiting the sampled area and strata were recorded.

Although we used *A. cucurbitina* and *S. globosum* for the functional response experiments, based on our previous knowledge on the abundance and persistence of these species within the crop, the field sampling scheme pointed *M. acalyph*a and *O. nigripalpis* as the most abundant species of orb-weavers and stalkers, respectively, during the raising of *P. spumarius*. Accordingly, it is reasonable to also consider the latter species as candidates for further experiments of functional response on *P. spumarius*. However, it must be taken into account that the sampling was carried out only at two groves and at the herbaceous strata. Thus, increasing the number of groves and vertical strata sampled may highlight further species or even confirm the relevance of our two formerly selected species.

For instance, *A. cucurbitina* is a widespread species present at three different vertical strata, the tree canopy, bushes, and the herbaceous layer, whereas *M. acalypha* prefers the herbs near the ground [30]. The importance of *A. cucurbitina* relies on the fact that both the spider species and *P. spumarius* inhabit the olive tree canopy [42,43,44], thus there occurs spatial overlapping of the potential natural enemy and the pest. However, spittlebugs disperse in late spring to non-cultivated patches that act as natural reservoirs [45]. In our study area, the most common surrounding patches correspond to semi-natural Mediterranean shrublands. *Synema globosum* is frequently observed in flowers of *C. ladanifer*, a common blossom found in these shrubland areas. Also, *S. globosum* is a wider spread species than *Runcinia grammica* Koch (1837) (Thomisidae), which was the most abundant recorded species of ambushers during the field sampling [30]. Accordingly, the selection of *S. globosum* relies on the fact that the species also inhabits the adjacent unmanaged habitats, this is, locals that could enhance the potential infection pressure on the focal field (i.e. olive groves) by pest migration [46].

Regarding the functional response found in laboratory for *A. cucurbitina* and *S. globosum* fed on *P. spumarius*, none of the curves reached the asymptote. This could be solved including higher initial prey densities; however, due to the limited volume of the arenas, an excessive prey density would be unreal (i.e., far away from a reasonable density in the field) thus making the results meaningless. No significant differences beyond the type of response were observed. This could be pointless at places with low densities of *P. spumarius* such as Spain [45]. However, the number of individuals of *P. spumarius* killed by each species could significantly differ at sites with high prey densities such as Italy [47], in which case the type-I response of *S. globosum* would be more efficient than that of *A. cucurbitina*. 

**Figure 4:**
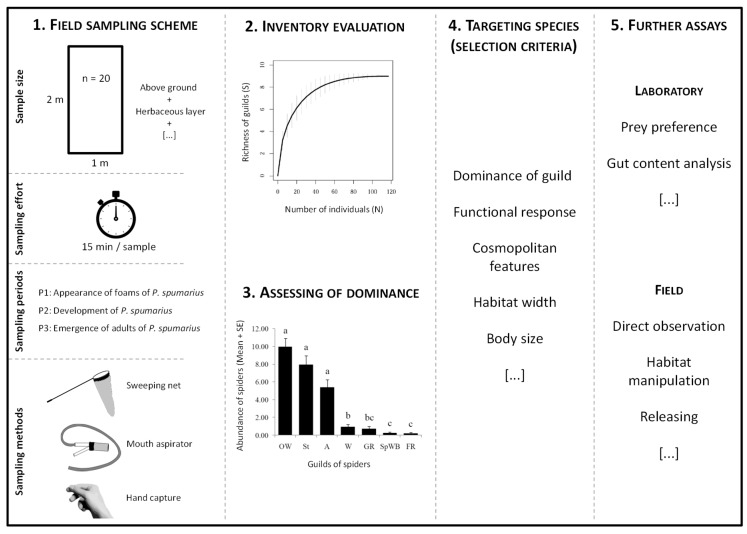
Workflow depicting the proposed protocol to select potential natural enemies of *Philaenus spumarius* among spiders in the olive grove.

Summarizing, the performed field sampling design followed by a guild-based faunistic inventory evaluation, the assessment of guild abundances during the outbreaks of *P. spumarius* populations, and the consideration of a few selecting criteria (e.g., guild dominance, cosmopolitan features, and functional response) allowed to define a protocol to deal with the whole workflow toward the selection of potential natural enemies among spiders to further work with in the absence of previous information (Figure 4).

Nevertheless, other aspect could also be considered. The criteria to select the predators to further work with could include additional parameters such as the body size. For example, although it was not initially considered, the body size of both the selected spider species was higher than the most abundant species. The body length of females of *A. cucurbitina* ranges from 4.5 to 9.5 mm and 5.5 to 6 mm for *M. acalypha*, whereas it ranges from 6.8 to 8 mm for *S. globosum* and 5.4 to 7.6 mm for *R. grammica* [30]. This fact could play a key role on hunting success both in terms of prey handling by the spider (e.g., manageability according to chelicerae size) [48] or the prey behavior (e.g., avoidance behavior) [49]. Moreover, since it would not be wise to assume that all spider species would follow the same functional response within the same guild, this still makes mandatory to test the functional response of different predators using the same hunting strategy to uncover both patterns and exceptions. Also, the effect of a resource-based competition and prey preference could have effect on the predatory efficiency and should be investigated, for example using multiple predators and prey species in functional response assays [29]. Finally, in terms of sampling design, targeting different vertical strata (e.g., trunk and canopy of the olive tree) most likely will improve the spectrum of potential species of candidate predators. Interestingly, Morente et al. [45] found that the sweeping was the most effective and least time-consuming method for sampling spittlebugs in the ground and canopy of the olive grove, so that the proposed protocol allows targeting potential natural enemies and pest monitoring at once. 

## 5. Conclusions

The proposed protocol aims to provide a time-saving, reproducible, and geographical context-free approach that could facilitate the targeting of a spider species as a potential natural enemy for subsequent research such as laboratory studies, direct observation in the field, and gut analytical methods toward the assessment of prey consumption, suitability for breeding, and releasing populations onto the crop (i.e., augmentation or inundation with natural enemies), as well as the design of habitat manipulation experiments within the biological conservation framework. 

## Figures and Tables

**Table 1:** Guilds, families, and species of Araneae identified in the complete pool of samples collected at the studied olive groves. Guild assignment follows Uetz et al. [23] and Cardoso et al. [31].

Guild	Family	Species	Abundance (N)
Ambushers	Philodromidae	*Philodromus albidus* Kulczyński, 1911	1
		*Philodromus* sp.	28
	Pisauridae	*Pisaura mirabilis* (Clerck, 1757)	6
	Thomisidae	*Runcinia grammica* (C. L. Koch, 1837)	73
		*Synema globosum* (Fabricius, 1775)	26
		*Thomisus onustus* Walckenaer, 1805	22
		*Xysticus cristatus* (Clerck, 1757)	1
		*Xysticus kochi* Thorell, 1872	12
		Immatures	339
Foliage runners	Cheiracanthiidae	*Cheiracanthium erraticum* (Walckenaer, 1802)	5
		*Cheiracanthium* sp.	8
	Sparassidae	*Micrommata ligurina* (C. L. Koch, 1845)	1
Ground runners	Dysderidae	*Dysdera crocata* C. L. Koch, 1838	2
	Gnaphosidae	*Haplodrassus rufipes* (Lucas, 1846)	4
		*Nomisia exornata* (C. L. Koch, 1839)	1
		*Nomisia* sp.	4
		*Zelotes* sp.	5
		Immatures	13
	Lycosidae	*Alopecosa albofasciata* (Brullé, 1832)	1
		*Arctosa villica* (Lucas, 1846)	2
		*Pardosa proxima* (C. L. Koch, 1847)	13
		Immatures	22
Orb-weavers	Araneidae	*Aculepeira ceropegia* (Walckenaer, 1802)	86
		*Agalenatea redii* (Scopoli, 1763)	6
		*Cyclosa algerica* Simon, 1885	1
		*Hypsosinga albovittata* (Westring, 1851)	3
		*Mangora acalypha* (Walckenaer, 1802)	201
	Uloboridae	*Uloborus walckenaerius* Latreille, 1806	10
Sheet web builders	Agelenidae	*Eratigena feminea* (Simon, 1870)	3
Space web builders	Dictynidae	Immatures	1
	Theridiidae	*Asagena phalerata* (Panzer, 1801)	3
		*Crustulina* sp. 1	1
		*Euryopis episinoides* (Walckenaer, 1847)	1
		*Phylloneta impressa* (L. Koch, 1881)	7
		*Simitidion simile* (C. L. Koch, 1836)	1
		Theridiidae sp. 1	2
		Theridiidae sp. 2	1
		Immatures	2
Specialists	Zodariidae	*Selamia reticulata* (Simon, 1870)	1
		*Zodarion* sp.	3
Stalkers	Oxyopidae	*Oxyopes heterophthalmus* (Latreille, 1804)	105
		*Oxyopes nigripalpis* Kulczyński, 1891	86
		*Oxyopes* sp.	276
	Salticidae	*Chalcoscirtus infimus* (Simon, 1868)	2
		*Heliophanus cupreus* (Walckenaer, 1802)	2
		*Icius hamatus* (C. L. Koch, 1846)	1
		*Pellenes brevis* (Simon, 1868)	1
		*Phlegra lineata* (C. L. Koch, 1846)	5
		Immatures	17
Wandering sheet/tangle weavers	Linyphiidae	*Agyneta* sp.	7
		Erigoninae sp.	62
		Linyphiidae sp. 1	1
		*Pelecopsis inedita* (O. P.-Cambridge, 1875)	31
		*Prinerigone vagans* (Audouin, 1826)	1
		*Styloctetor romanus* (O. P.-Cambridge, 1873)	1
		*Tenuiphantes tenuis* (Blackwall, 1852)	6
		*Typhocrestus bogarti* Bosmans, 1990	2
		Immatures	37
Araneae immatures (not identified)			13
Total			1578
